# Toward Measuring Target Perception: First-Order and Second-Order Deep Network Pipeline for Classification of Fixation-Related Potentials

**DOI:** 10.1155/2020/8829451

**Published:** 2020-11-19

**Authors:** Hong Zeng, Junjie Shen, Wenming Zheng, Aiguo Song, Jia Liu

**Affiliations:** ^1^State Key Laboratory of Bioelectronics, Jiangsu Key Lab of Remote Measurement and Control, School of Instrument Science and Engineering, Southeast University, Nanjing 210096, China; ^2^CICAEET, Nanjing University of Information Science and Technology, Nanjing 210044, China; ^3^Key Laboratory of Child Development and Learning Science (Ministry of Education), School of Biological Sciences and Medical Engineering, Southeast University, Nanjing 210096, China

## Abstract

The topdown determined visual object perception refers to the ability of a person to identify a prespecified visual target. This paper studies the technical foundation for measuring the target-perceptual ability in a guided visual search task, using the EEG-based brain imaging technique. Specifically, it focuses on the feature representation learning problem for single-trial classification of fixation-related potentials (FRPs). The existing methods either capture only first-order statistics while ignoring second-order statistics in data, or directly extract second-order statistics with covariance matrices estimated with raw FRPs that suffer from low signal-to-noise ratio. In this paper, we propose a new representation learning pipeline involving a low-level convolution subnetwork followed by a high-level Riemannian manifold subnetwork, with a novel midlevel pooling layer bridging them. In this way, the discriminative power of the first-order features can be increased by the convolution subnetwork, while the second-order information in the convolutional features could further be deeply learned with the subsequent Riemannian subnetwork. In particular, the temporal ordering of FRPs is well preserved for the components in our pipeline, which is considered to be a valuable source of discriminant information. The experimental results show that proposed approach leads to improved classification performance and robustness to lack of data over the state-of-the-art ones, thus making it appealing for practical applications in measuring the target-perceptual ability of cognitively impaired patients with the FRP technique.

## 1. Introduction

The topdown determined visual object perception refers to the ability of a person to identify a prespecified target in view based on visual input [1]. Subjects suffering from Alzheimer's disease (AD) generally have difficulties in distinction between target and nontarget. An objective and effective way for early assessing such a functional deficit in suspected AD patients could be examining the brain responses, i.e., event-related potentials (ERPs), acquired by an electroencephalographic (EEG) device, during the engagement of human subjects in a visual perception task [2, 3]. In specific, the P300 ERPs are usually used to infer whether the subject is looking at a target or not, without the need for overt conscious behavioral/verbal reports by the subject. Common practice in lab-grade P300 research experiments is that the subject is asked to restrict the eye movements by fixating his/her eyes on a fixed space for avoiding ocular artifacts, while stimuli are presented in an oddball paradigm. However, in real-world P300 applications, it is difficult for keeping uncooperative AD patients maintain their eyes fixated on the fixed space after each stimulus onset, which is unnatural and easily leads to quit of tasks. A technologically more advanced approach that has gained popularity in recent years is the simultaneous recording of eye movements and EEG signals during the viewing of stimuli. In such coregistration studies, the EEG signals can then be aligned to the subject self-generated fixation onset, yielding fixation-related potentials (FRPs) [4, 5]. Moreover, it has already been revealed that FRPs following fixation of a target are different than FRPs following fixation of a nontarget [6]. All in all, compared to traditional ERP studies without eye movements, the FRP technique offers AD patients the opportunity to conduct tasks that embrace rather than limit eye movements, opening up the possibility of practical applications for target-perceptual ability measurement by analyzing brain responses in a visual search task.

Since the raw FRPs are multichannel time series possessing a strong underlying structure and a complex distribution, the measurement of target-perceptual ability highly depends on a good feature representation for FRPs, which should discriminate between the fixation onto target and the fixation onto nontarget from single-trial FRPs. Thereby, this issue is generally formulated as the feature extraction or feature learning problem for single-trial classification of FRPs. Early FRP studies [7–9] often directly utilize the raw temporal form concatenated into a vector as the feature for a single-trial FRPs, whereas some latter efforts [10–12] have applied spatial filters (e.g., PCA and xDAWN [13]) on FRPs, in order to obtain a representation with higher signal-to-noise ratio (SNR) and thus improved classification performance. Although the majority of FRP-based systems still rely on the manually extracted features, recent works have explored the application of deep learning [14]. In particular, the convolutional neural networks (CNNs) introduce the nonlinearity and hierarchy into the feature extraction, by learning local nonlinear features (through convolutions and nonlinearities) and representing higher-level features as compositions of lower level features (through multiple layers of processing), having achieved state-of-the-art performance [15, 16]. Since all the shallow feature extraction methods and the deep CNNs mentioned above capture only first-order statistics (e.g., mean and maximum) from the input FRP trials, we call them first-order models.

To characterize the complex underlying structure of ERPs, recent endeavors have also resorted to extract second-order statistics. The second-order statistics such as sample covariance matrix estimated with input ERP trials allows to capture relevant information (e.g., correlations) for groups of channels, whose temporal structures show high statistical dependency among them [17, 18]. Besides, the representational richness of covariance is also rooted in its Riemannian manifold structure. Namely, the covariance matrix is symmetric positive definite (SPD), lying on the curved Riemannian manifold instead of the Euclidean space. By taking the Riemannian geometry of the input covariance into account, several ERPs studies have developed shallow Riemannian models, such as minimum distance to Riemannian mean (MDRM) [19] and tangent space linear discriminant analysis (TSLDA) [19]. They have yielded better classification performance than methods that ignore the geometry information by simply reshaping the covariance matrix into a vector. Moreover, our previous work [20] applies the recently proposed deep Riemannian model SPDNet [21], which is similar in architecture to CNNs but with layers designed for SPD matrix, to the single-trial FRPs classification problem. The results show that it leads to advantageous performance over the shallow Riemannian models [20]. As both the shallow and deep Riemannian models deal with the covariance of data, they are called second-order models.

Up to now, it is not difficult to notice the following possible limitations of current efforts exploring deep neural network (DNN) techniques for classifying FRPs. On one hand, the CNN-based approaches have ignored the exploiting of second-order statistics such as correlations among feature maps from convolutional layers. On the other hand, the input covariance matrix has been confined to the one computed from raw signals of low SNR in SPDNet-based study. To this end, we propose a novel feature representation learning model within DNN framework. More specifically, to extract the discriminative and robust representation for single-trial classification of FRPs, the encoding of first-order and second-order statistical information of data is performed sequentially, i.e., using a low-level fully temporal convolution and then a high-level deep Riemannian geometric processing. Furthermore, catering to the FRP classification problem, we propose a new midlevel second-order pooling layer to bridge the low-level convolution and high-level SPD representation learning, where the convolutional features are aggregated across time into an SPD matrix of a rich representation capability. In particular, both the low-level convolution subnetwork and the midlevel pooling layer are designed to preserve the temporal ordering of the FRP dynamics, which is known to be crucial for classifying FRPs. We evaluate the performance of the proposed model on FRP datasets collected from a guided visual search task. The experimental results show that the proposed approach leads to improved classification performance and robustness to data scarcity (by reducing the size of the dataset to only 25% of its original size) over the state-of-the-art DNNs. In addition to the role of the overall model architecture, the model ablation study is also performed to validate our temporal ordering persevering designs for pipeline components. The results presented in this paper are the first step toward practical applications in measuring the target-perceptual ability of suspected AD patients with FRP techniques.

In summary, the contribution of our paper is two-fold:

1. For the problem of single-trial FRP classification, the present study is, to the best of our knowledge, the first to introduce a deep learning pipeline involving a low-level convolution subnetwork followed by a high-level Riemannian manifold subnetwork, with a novel midlevel pooling layer bridging them. The proposed pipeline goes beyond the existing models consisting of first-order only or second-order only subnetworks, yielding more discriminative and more robust feature representations.
2. We propose the temporal ordering persevering designs for both the low-level first-order convolution subnetwork and the midlevel second-order pooling layer. Such designs allow to capture intrinsic temporal information of FRP dynamics, being in favor of the single-trial classification of FRPs.

The remainder of our paper is organized as follows.  Section 2 gives the description of the collected FRP dataset in a guided visual search task for validating our proposed model.  Section 3 illustrates the details of the proposed model. The performance evaluation settings are introduced in Section 4, followed by extensive results obtained on various amounts of data presented in Section 5. We discuss our findings in Section 6. Finally, a conclusion is provided in Section 7.

## 2. Materials

### 2.1. Participants

There were 4 female and 6 male volunteers from the Southeast University, with normal or corrected to normal vision, no report of eye or neurological diseases, and ages ranging from 21 to 26 yr (median of 23 yr), participated the study. The experimental procedure and written consent form for this study were approved by the ethics committee at the Southeast University, and adhered to the ethical standards of the sixth revision of the Declaration of Helsinki. All participants gave their informed written consent to participate in the study.

### 2.2. Guided Visual Search Task

**Figure 1:**
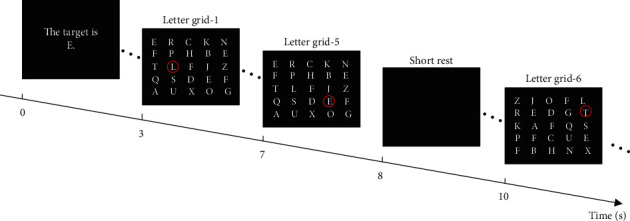
The procedure of the guided visual search task.

Participants were asked to perform a guided visual search task on a 5 × 5 grid of equiluminant, equally spaced 25 capital English letters (i.e., *A*, *B*, *C*, *D*, *E*, *E*, *E*, *F*, *F*, *F*, *G*, *H*, *J*, *K*, *L*, *N*, *O*, *P*, *Q*, *R*, *S*, *T*, *U*, *X*, and *Z*) of 2° visual angle in white presented on a background in black (see Figure 1). The task was performed in 4 blocks for each subject. Before the presence of grid letters for each block, the prompt for the designated target (either “*E*” or “*F*”) was displayed on the screen for 3 seconds. During the display of grid letters, eye fixations were guided across the grid by a red circle (3.8° visual angle) that randomly surrounded one of the letters. Such a guiding circle remained visible for one second (i.e., a trial) after which it moved to a different letter location. Participants were instructed to saccade to and fixate on the letter in the center of the red circle for 1 second and press the space bar of a keyboard with their forefinger of the right hand only when the visual target was present (for keeping subjects engaged in the task). A 2-second short rest gap and a 10-second long rest gap displaying a blank screen in black were intersected between every 5 movements and 25 movements of the guiding circle, respectively. The grid of letters was updated every 5 movements of the guiding circle by randomly changing the locations of the 25 letters. A single block of guided visual search task consisted 250 movements of the red circle, lasting about 10 minutes. A 5-miniute rest period was inserted between each block. Since there were always 3 target letters out of the 25 letters for each grid, the target appearance probability is 3/25 = 12% in each block. The presentation program was developed with Matlab Psychtoolbox, and the grid was displayed on Dell 1280 × 1024 LCD monitor with the refresh rate at 60 Hz.

### 2.3. Eye Movements and EEG Recordings

**Figure 2:**
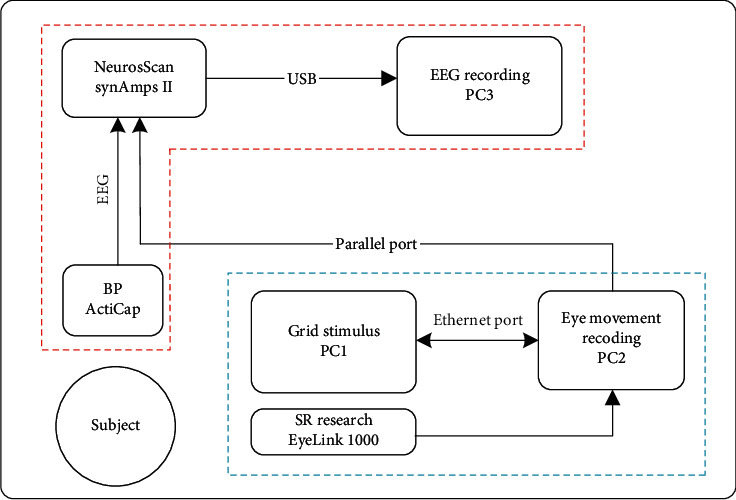
The experimental setup of the guided visual search task.

The experimental setup of the guided visual search task is depicted in Figure 2. Eye movements were recorded with an EyeLink 1000 system (SR Research, Ontario, Canada). The eye tracker worked in monocular mode, where it recorded the subjects' dominant eye with the sampling rate of 1000 Hz. For moderate head movement reduction, subjects were positioned in a chinrest at a distance of 57 cm from the LCD monitor. The nine-point calibration was conducted prior to the experimental blocks, and drift corrections were performed every 25 movements of the red guiding circle (trials) during the experiments.

The EEG signals were sampled at 1000 Hz with a 64-channel 10–20 montage active electrode cap (ActiCap, BrainAmp, BrainProducts, Munich, Germany) and an EEG amplifier (SynAmps II, Neuroscan, Compumedics, Victoria, Australia). Signals collected from 19 electrodes (F*z*, FC*z*, C*z*, C3, C4, CP*z*, P*z*, P3, P4, P7, P8, PO*z*, PO3, PO4, PO7, PO8, O*z*, O1, and O2) were referenced to the left mastoid, and the ground electrode was placed on the forehead. EOG electrodes were sticked to the outer canthi of dominant eye, as well as below the eye. All the electrodes impedance was kept below 10 kΩ during the experiments.

Data from the stimuli presentation, EEG, and eye-tracking acquisition systems were synchronized using the custom software during the experiments. In specific, the stimuli presentation computer sent an event message to the eye tracker host computer through the Ethernet port, once the red guiding circle moved to a new letter; the eye tracker host computer then transmitted a TTL trigger to the EEG amplifier via the parallel port. The synchronization lag between EEG and eye-tracking acquisition systems was about 1 ms, which is neglectable.

### 2.4. Generation of FRP Datasets

The single-trial FRPs were then extracted from the synchronized EEG and eye-tracking recordings with the following steps implemented in Matlab (Release 2014b). Firstly, the fixation onset in the raw eye-tracking data was detected using three thresholds: velocity (30°/*s*), acceleration (8000°/*s*^2^), and saccadic motion (0.15°) [22]. Moreover, only valid fixations were kept in the analysis, which were the first exact fixations without blinking landed on a circular area of 3° visual angle from the center of the guiding circle and lasted longer than 350 ms [23]. Secondly, EEG signals were filtered using a second-order zero-phase Butterworth filter with a 1–40 Hz passband. Furthermore, although the eye remained relatively stable during a fixation, there were still micro eye movement-related artifacts that contaminated the neural response. Infomax independent component analysis (ICA) was then performed to identify sources of ocular artifact, and the independent components were rejected based on their covariance with the eye-tracking data (components with a ratio of 1.1 or greater were removed) [24]. Thirdly, EEG signals were aligned to fixation onset and segmented between −200 ms and 1000 ms from the fixation onset. The baseline correction were applied to each epoch in the time window (–200–100) ms from fixation onset. Furthermore, single-trial FRPs in the time window (01000) ms were used for the latter analysis. Next, we rejected fixation-locked trials if peak-to-peak activity surpassed a 100 *μ*V threshold using a window size of 50 ms and a sliding step of 25 ms. The visual inspection of the EEG signals confirmed that artifacts were clearly reduced. Lastly, EEG data were downsampled at 128 Hz and labeled according to fixations on target or nontarget. The resulting FRP dataset for each subject is summarized in Table 1.

## 3. Methods

Firstly, the recently proposed first-order deep model EEGNet [16] and the second-order deep model SPDNet [21], based on which our work is developed, are briefly reviewed. They consist of first-order only and second-order only subnetworks, respectively. Afterward, we describe the proposed approach that builds the first-order and second-order subnetworks in sequence, aiming at better capturing relevant statistics for the single-trial classification of FRPs.

### 3.1. EEGNet

**Figure 3:**
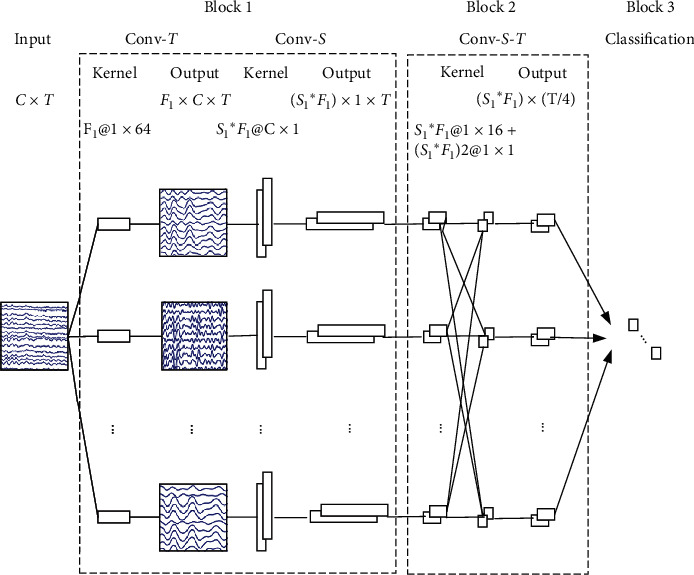
The architecture of EEGNet-*F*_1_, *S*_1_ model (*F*_1_ and *S*_1_ represent the number of temporal filters and spatial filters in convolutional block 1, respectively). Conv-T, Conv-S, and Conv-S-T denote the temporal, spatial, and spatiotemporal convolution, respectively. The batch normalization, average pooling, dropout, and activation layers are not depicted for clarity. The size of the output is the one from either the convolution or the activation.

Due to the physical origin of the noninvasive EEG, there would be no obvious hierarchical spatial local and global modulations [15]. By contrast, EEG is consistently found to be organized across multiple time scales [25], indicating that there are temporal hierarchies of local and global modulations. Based on the facts stated above, EEGNet has been proposed in [16], which is a compact convolutional neural network tailored to the classification of EEG signals. It takes the raw temporal form as input, i.e., the 2D-array of size *C* × *T*, for *C* channels and *T* time instances. The EEGNet architecture is visualized in Figure 3, consisting of three blocks. The first block performs temporal convolutions (Conv-T in Figure 3) to mimic bandpass frequency filters, followed by depthwise spatial convolutions (Conv-S in Figure 3) that act as spatial filters. Such depthwise spatial convolutions are not fully connected to all the previous layers, so as to learn spatial filters for each temporal filter, which together improve SNR and reduce the number of trainable parameters to fit. The second block involves depthwise temporal convolution to individually summarize each local, low-level feature map from the previous block in a larger time scale, followed by pointwise convolutions to optimally merge the outputs afterward for capturing global and high-level features. The third block directly passes the flattened features to a softmax classification with *K* units (*K* is the number of classes in the data). Each convolution layer is followed by batch normalization. There is no activation function between the two convolution layers within a block, but the exponential linear unit (ELU) nonlinearity is employed between blocks, followed by 2D average pooling and dropout layers. In a word, by performing linear combinations, average pooling, and elementwise nonlinear operations, EEGNet can be thought of as extracting solely first-order statistics from FRP trials.

### 3.2. SPDNet

**Figure 4:**
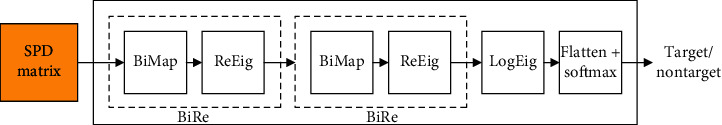
The architecture of SPDNet with two BiRe blocks.

The SPD matrices reside on the curved Riemannian manifold *𝒮*^+^, and thus, it leads to loss of geometric information and poor results by directly flattening and applying classification models devised in Euclidean space. SPDNet is a deep learning model for SPD matrix as input, which learns the nonlinear transformation projecting an SPD matrix into a more discriminative one [21]. In specific, an input matrix **X**_*l*−1_ ∈ *𝒮*_*d*_*l*−1__^+^ at the layer yields **B**_*l*_ ∈ *ℝ*^*d*_*l*_×*d*_*l*_^ via a bilinear mapping (the BiMap layer), which in turn yields **X**_*l*_ ∈ *𝒮*_*d*_*l*__^+^ at the layer *l* through the nonlinear activation, i.e., rectified eigenvalues (ReEig layer) activation, by the following steps:

(1) $$\begin{array}{c} B_{l}=W_{l}^{⊤}X_{l-1}W_{l}\text{ with }W_{l}\in ST^{d_{l-1}\times d_{l}},\\ X_{l}=U_{l}\max\left(\Lambda_{l},\epsilon I\right)U_{l}^{⊤}\text{ with }B_{l}=U_{l}\Lambda_{l}U_{l}^{⊤}, \end{array}$$

where *𝒮𝒯*^*d*_*l*−1_×*d*_*l*_^ is the compact Stiefel manifold of semiorthogonal rectangular matrices, **B**_*l*_ = **U**_*l*_Λ_*l*_**U**_*l*_^⊤^ represents the eigenvalue decomposition of **B**_*l*_, and *ϵ* is a small positive threshold for the eigenvalues. The final LogEig layer endows elements in Riemannian manifold with a Lie group structure so that matrices can be flattened and conventional Euclidean classifications can be applied. The BiMap and ReEig layers are used together as a block (abbreviated as BiRe), and such BiRe blocks can be repeated as many as wanted, to achieve a high-performance deep learning model.  Figure 4 depicts the architecture of SPDNet with two BiRe blocks as the feature extractor and one LogEig layer on the top. The covariance matrix estimated from single-trial FRPs is SPD, and thus, SPDNet could be applied on such SPD matrices to deeply learn the discriminative second-order statistics from the single-trial FRPs.

### 3.3. The Proposed Classification Pipeline

**Figure 5:**
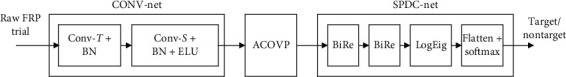
The pipeline of the proposed model. BN and ELU stand for batch normalization and ELU activation, respectively.

The pipeline of the proposed model is shown in Figure 5, consisting of three stages. FRPs are known to suffer from external noises in the environment and internal interfering cerebral activities that are irrelevant to the brain responses to fixations on targets. Due to this, minimally processed time-series single-trial FRPs are fed into the first convolutional block of EEGNet (denoted as CONV-Net in Figure 5), which performs multiple-frequency-specific spatial filtering on the raw EEG. Next, we propose to conduct augmented covariance pooling on the convolutional feature maps, for obtaining an SPD matrix as a powerful representation (referred to as ACOVP in Figure 5). Lastly, the manifold network SPDNet is employed to deeply learn the second-order statistics and for the classification (denoted as SPDC-Net in Figure 5). The details of each stage for the proposed model are explained below.

#### 3.3.1. Fully Temporal Convolutional Subnetwork (CONV-Net)

The temporal structure of the amplitude changes within a trial has been found to be important for detecting target-related ERPs [16]. Recall that the fully temporal convolutional layers in the first block of EEGNet facilitate the extraction of frequency-specific, spatially localized time-series representation of FRPs, while EEGNet further utilizes another block of convolutional layers to summarize such features into global high-level descriptive representation for the task. It indicates that the first block of EEGNet preserves the temporal structure within raw FRP trials, whereas such temporal information may be largely lost for the second block. Therefore, to build a powerful SPD matrix as a temporal representation of FRPs for further extracting second-order statistics, instead of the output feature maps from activation functions of the second block, those from activation functions of the first block in EEGNet are utilized. In specific, we learn 8 temporal filters of size 1 × 64 and further 2 spatial filters of size *C* × 1 (*C* = 19 channels) per temporal filter, as it has been shown to perform well for P300 classification in [16].

#### 3.3.2. Augmented Covariance Pooling Layer (ACOVP)

To deeply learn the second-order information, we introduce the augmented covariance pooling after fully temporal convolutional layers. Given a single-trial EEG, let the activation map of the fully temporal convolutional layers be a *D* × *H* × *W* tensor with feature channel *D* (*D* = 16, i.e., output of 8 temporal filters and 2 spatial filters per temporal filter), height *H* (*H* = 1), and width *W* (*W* = 128, i.e., the no. of time instances in a single convolutional feature map). We reshape it into a matrix **X**_*i*_ ∈ *ℝ*^*D*×*N*^ with *N* = *H* × *W* and further center it along row, and the centered matrix is denoted as ${\bar{X}}_{i}$. We then perform the covariance pooling by estimating the sample covariance matrix as

(2) $$\begin{array}{c} C_{i}=\frac{1}{N-1}{\bar{X}}_{i}{\bar{X}}_{i}^{⊤}. \end{array}$$

However, if the time instants within a trial was randomly shuffled, the estimate of its covariance matrix does not change. In other words, using the covariance matrix for the classification would probably lead to the loss of the precise temporal information carried in learned convolutional features. To effectively capture the temporal ordering of convolutional features within a trial, we calculate the augmented covariance matrix by following the ERP template concatenation method suggested in [26]. Specifically, an augmented trial ${\tilde{X}}_{i}\in {\mathbb{R}}^{2D\times N}$ is built by concatenating ${\bar{X}}_{\text{tar}}$ and ${\bar{X}}_{i}$:

(3) $$\begin{array}{c} {\tilde{X}}_{i}=\left[\begin{array}{c} {\bar{X}}_{\text{tar}}\\ {\bar{X}}_{i} \end{array}\right], \end{array}$$

where ${\bar{X}}_{\text{tar}}$ stands for the centered **X**_tar_, i.e., the prototyped target FRP response, obtained by averaging trials in the learned convolutional representation from the target class:

(4) $$\begin{array}{c} X_{\text{tar}}=\frac{1}{\left|K^{+}\right|}\sum_{X_{i}\in K^{+}}X_{i}, \end{array}$$

and *𝒦*^+^ designates the group of target FRPs trials. The augmented covariance pooling is thus carried out by

(5) $$\begin{array}{c} AugC_{i}=\frac{1}{N-1}{\tilde{X}}_{i}{\tilde{X}}_{i}^{⊤}, \end{array}$$

where **AugC**_*i*_ ∈ *ℝ*^2*D*×2*D*^. In our experiments, we found that augmented covariance **AugC**_*i*_ is always SPD since more samples are observed than their dimension (2*D* = 2 × 16 is less than *N* = 128). Even if the matrices are only positive semidefinite, they can be made SPD by adding small positive constants to the diagonal entries of the augmented covariance matrix:

(6) $$\begin{array}{c} AugC_{i}^{+}=AugC_{i}+\lambda \text{trace}\left(AugC_{i}\right)I, \end{array}$$

where *λ* is a small positive constant and **I** is the identity matrix.

In essence, the obtained SPD matrix **AugC**_*i*_ acts as a midlevel representation bridging the local low-level fully temporal convolutional features and the global high-level task-specific features.

#### 3.3.3. SPD Matrix Learning and Classification Subnetwork (SPDC-Net)

We further perform the end-to-end learning on the SPD matrix **AugC**_*i*_ with SPDNet. Considering the size of the SPD matrix transformation 32 × 32 is not big enough, an SPDNet architecture consisting of two BiRe blocks without dimension reduction (i.e., *d*_*l*_ ≡ *d*_*l*−1_ in equation (1)) is adopted in this paper, according to the preliminary experiments on the training set. The two BiRe blocks are followed by a LogEig layer for mapping into Euclidean space, a flatten layer, and a softmax layer for classification.

## 4. Experiments

### 4.1. Implementation Details

The output sizes of each stage in the pipeline are given in Table 2. To build the CONV-Net, we use the EEGNet source code provided by the authors in [16] ^1^. The EEGNet-8, 2 model (8 temporal filters followed by 2 spatial filters per temporal filer) is trained on NIVIDA GTX 1060ti GPU in TensorFlow [27], using Keras API [28]. To handle the imbalanced data issue in our problem (there is a 6803 : 1015 odds between nontargets and targets, see Table 1), a class-weight is applied to the loss function. Specifically, the class-weight applied is the inverse of the proportion in the training data, with the majority class set to 1. The Adam algorithm with default parameter settings [29] is used to optimize the class-weighted cross-entropy loss function. The dropout probability is 0.25 for all layers. We train the model for 100 iterations and perform early stopping, saving the model weights producing the lowest validation loss.

The fully temporal feature maps for each training trial are then loaded for building the augmented covariance matrix of size 32 × 32 in the ACOVP layer. The SPDC-Net is implemented based on the Matlab version source code of SPDNet in the original paper [21], with defaulted parameters on an Intel 3.2 G Hz Core i5 PC with 12 GB of RAM and Windows 10 operating system^2^. Again, the class-weight is applied to the cross-entropy loss function. The stochastic gradient descent algorithm on Riemannian manifolds is exploited to train the network. We train SPDC-Net for 100 iterations, and the validation stopping strategy is adopted again.

### 4.2. Comparison between Existing Methods and Ours

The approaches that either extract the first-order or the second-order statistics are compared with our method. These compared methods are briefly introduced below.

#### 4.2.1. First-Order Models

1. PCA + LDA [11]: Finke et al. [11] first concatenate the channelwise FRP trials to form a high-dimensional vector, and then apply principal component analysis (PCA) to reduce the dimensionality (99.9% of variance was kept). Finally, the classification is achieved using Fisher's linear discriminant analysis (LDA). The method denoted as PCA + LDA has been implemented in *Python* using the scikit-learn library [30].
2. xDAWN + LDA [10]: xDAWN is a commonly used spatial filtering algorithm for enhancing ERP [13]. xDAWN is implemented in *Python* using the MNE library [31] with the suggested parameter in its original paper [13].
3. DeepConvNet [15]: there are four convolutional-max-pooling blocks in DeepConvNet, where the structure of the first block is similar to that of the first block of EEGNet. The ShallowConvNet proposed by the same authors in [15] has not been compared because it is tailored for oscillatory EEG signal (e.g., motor imagery) classification. We have adopted the source code (within TensorFlow framework) for DeepConvNet reimplemented by the authors in [16] with default parameter settings^3^.
4. EEGNet [16]: the same EEGNet-8, 2 model is compared, and its training configuration is the same as for our model.

#### 4.2.2. Second-Order Models

1. MDRM [19]: the MDRM classifier computes a geometric mean for each class using training SPD data and then assigns an unlabeled trial in the SPD representation to the class corresponding to the closest mean. It has been implemented in *Python* using the MNE library [31].
2. TSLDA [19]: this model projects the input SPD matrix to a tangent space followed by an LDA classifier. It has been implemented in *Python* with the MNE library [31].
3. SPDNet [21]: the same SPDNet-2BiRe model (2 BiRe blocks) is compared, and its training configuration is the same as for our model.

For the second-order models above, the ERP template concatenation strategy [26] has also been adopted to build the augmented covariance matrix using the raw EEG trial. Besides, no particular processing is adopted for the LDA classifier-based methods (i.e., PCA + LDA, xDAWN + LDA, and TSLDA) and the Riemannian distance based method (MDRM) regarding to the imbalanced data issue, since there is no reliable evidence to support the claim that an imbalanced data set has a negative effect on the performance of LDA [32], nor MDRM [19].

The performance of the single-trial FRP classification models is evaluated in a 5-fold chronological cross-validation (CV) using subject-specific data. For the training and test data split through the cross-validation procedure, the target-to-nontarget ratio has been kept the same as in the full data. Furthermore, randomly selected 20% data from the training set are used for model validation.

We assess the performance of classifiers using the area under curve (AUC) measure. The AUC measure is chosen because it is not effected by the prior class probability as is the classification accuracy measure, which is only meaningful when both classes are balanced in the test set. The Friedman test is conducted to assess the effect of different models on AUC. Post hoc analysis is conducted with Fisher's least significant difference (LSD) correction. The significant level is set at 0.05.

In order to study the robustness of models to the lack in data volume (as it could happen in real applications), we further report results on reduced sets of data, with the best first-order model and the best second-order model among the compared ones on full sets of data, as well as our proposed one. Specifically, keeping the target-to-nontarget ratio, the same as in the full set, 50% and 25% trials are randomly selected for each subject, respectively. Such random selections have repeated 10 times, resulting in 10 reduced sets consisting 50% and 25% of full set, respectively. For such two types of reduced sets, the 5-fold cross-validation classification is conducted per reduced set, and then the mean and standard deviation of 5-fold cross-validation AUC across 10 reduced sets and subjects are reported. In the statistical analysis, the averaged 5-fold cross-validation AUC across 10 reduced sets for each subject acts as the repeated measure.

### 4.3. Comparison between Different Components for Our Model

Through the model ablation analysis, we further examine the influence of different pipeline components of our model on its performance. Specifically, different design choices for CONV-Net/ACOVP are tested while keeping other components unchanged. Besides, the training configurations for different design choices are kept the same. The effects of different design choices are investigated by comparing the 5-fold CV averaged AUC of resulted models on full set of subject-specific data. The Wilcoxon sign rank test is applied for evaluating the statistical significance of the performance difference between two design choices.

## 5. Results

### 5.1. Grand Average Analysis of FRPs

**Figure 6:**
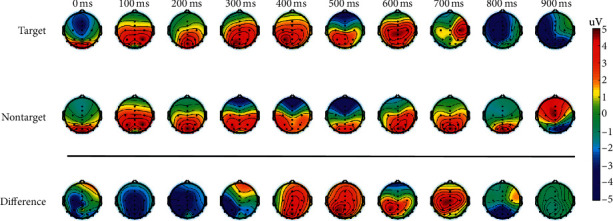
Topographical maps for the guided visual search task. The neural correlates of target detection have generally appeared after around 250 ms with a strong P300 component only for the fixations on targets. Time “0” ms in the topographical plots corresponds to fixation onset.

**Figure 7:**
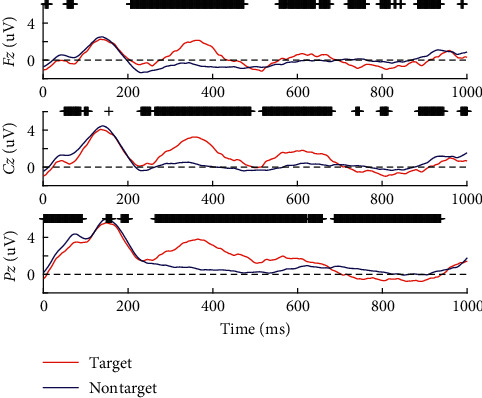
Grand average over all ten participants for target and nontarget FRPs. Targets have generally elicited a clear P300 component in the guided visual search task. The significantly different time instances between target and nontarget are marked by “+” (Wilcoxon rank-sum test, *p* < 0.05 corrected for multiple comparisons).

Figures 6 and 7 show the time-evolved topographical maps and the grand average (Fz, Cz and Pz) over all ten participants for target and nontarget FRPs during the guided visual search task, respectively. From such two figures, it can be observed that only fixations to targets elicit a sustained positive P300 component starting from around 250 ~ 300 ms, with a centroparietal topography. Moreover, in general, there is a clear difference in the amplitude of FRPs between fixations to target and nontarget in channel Fz, Cz, and Pz around 300 ~ 500 ms (Wilcoxon rank-sum test with a false discovery rate correction for multiple comparisons).

### 5.2. Comparison Results between Existing Methods and Ours

#### 5.2.1. Results on Full Set of Data

The 5-fold cross-validation AUC measures for all the methods on full set of data from 10 subjects are shown in Table 3. Different methods have significant influence on the performance. For the first-order models, the deep models, i.e., DeepConvNet and EEGNet, significantly outperform the conventional shallow models, i.e., PCA + LDA and xDAWN + LDA (LSD correction of 6, *p* < 0.05), while not performing significantly different between themselves. Regarding to the second-order models, the deep model, i.e., SPDNet, has achieved significantly superior performance to the shallow models, i.e., MDRM and TSLDA (LSD correction of 3, *p* < 0.05). The optimal result (0.9317 ± 0.0236) is obtained with our model, and it has obtained the highest averaged AUC for 9 out of 10 subjects. The performance difference between our method and the state-of-the-art first-order models (DeepConvNet and EEGNet), and that between our method and the state-of-the-art second-order model SPDNet are statistically significant (LSD correction of 6, *p* < 0.05). In addition, we observe that the deep models generally show smaller standard deviation of AUC across subjects than shallow models, and our model achieves the lowest one among all the methods, suggesting our method being more robust to subject-dependent differences than other methods. All these results indicate that our method provides more reliable classification performance, which may be due to the reason that our model benefits from building the first-order and second-order subnetworks in sequence.

#### 5.2.2. Results on Reduced Set of Data

**Figure 8:**
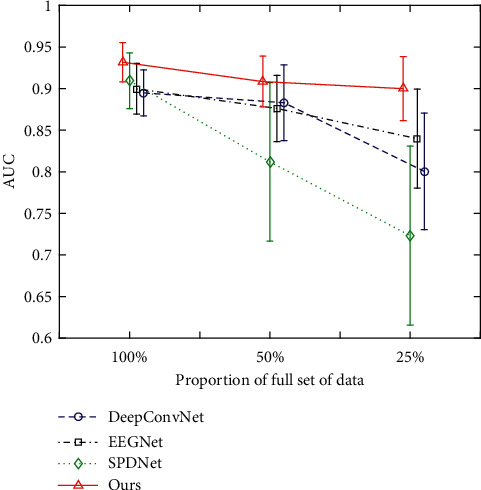
Performance (5-fold CV AUC) comparison on reduced sets of data.

Figure 8 depicts the performance of the best models (i.e., DeepConvNet and EEGNet) among compared first-order ones, the best model (i.e., SPDNet) among compared second-order ones, and our model with decreasing amount of data. In fact, our model consistently outperforms DeepConvNet, EEGNet, and SPDNet with statistical significance (LSD correction of 6, *p* < 0.05) across different amounts of data. Besides, there are two remarks regarding the results shown in Figure 8. First, as the number of training data decreases, the performance of SPDNet deteriorates the most fast among the four methods. It could be attributed to the fact that SPDNet takes the augmented covariance matrix built with the raw EEG trial as the input, suffering from a poor template estimation due to insufficient target trials and the low SNR of them. In contrast, our model builds the midlevel SPD representation with convolutional features of higher SNR. It thus may contribute to amplifying the interclass difference, leading to significantly upgraded robustness of the second-order deep learning through SPDNet in the presence of limited data. Second, the performance of DeepConvNet and EEGNet drops less fast than SPDNet, but has still degraded faster than our model. Moreover, even with 25% of full set of data, the performance of our model (0.8999±0.0384) is still comparable to that of DeepConvNet (0.8948±0.0277) and EEGNet (0.8997±0.0305) with full set of data. Possible explanations for this discrepancy are given below. Although the fully temporal first-order convolution is leveraged to enhance the SNR in the first network block in DeepConvNet, EEGNet, and our method, the latency variability in raw FRPs across trials has not been handled yet. Therefore, our method further performs deep second-order covariance learning on Riemannian manifold in subsequent network blocks. In particular, the midlevel augmented covariance representation encodes the correlation of local convolutional temporal features in a translationally invariant way. Besides, the FRP template obtained by averaging across trials is utilized for building the augmented covariance, which also contributes to reduce the model sensitivity to the latency variability across trials. In other words, the second-order deep network following the fully temporal convolutional network (i.e., our model) explicitly helps to reduce the intraclass variations, whereas the first-order deep convolutional network following the fully temporal convolutional network (i.e., DeepConvNet and EEGNet) implicitly addresses such an issue. Explicitly handling the intraclass variations may effectively reduce the model complexity, resulting in superior robustness of our method to DeepConvNet and EEGNet in case of limited data.

#### 5.2.3. Data Visualization

**Figure 9:**
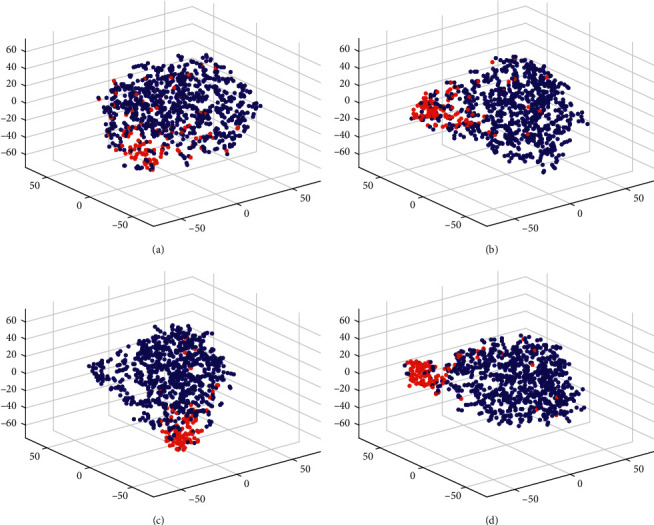
t-SNE of 100% raw data and outputs from each layer of our model. (a) Raw data; (b) outputs from CONV-Net; (c) outputs from ACOVP layer; (d) outputs from SPDC-Net. Red and blue dots represent the target and nontarget trials, respectively.

**Figure 10:**
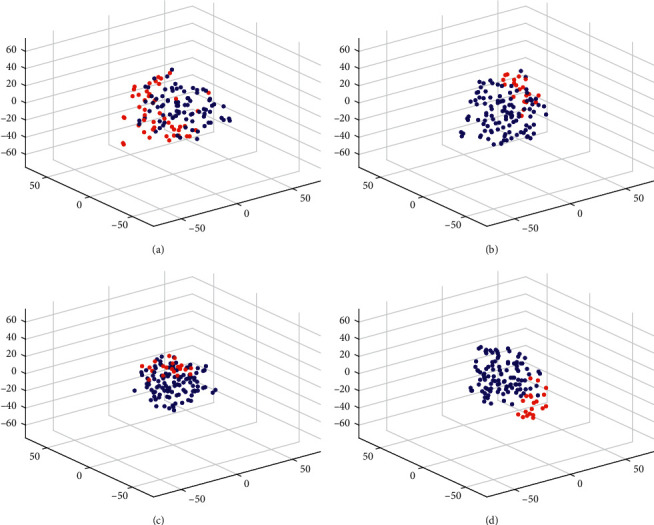
t-SNE of 25% raw data and outputs from each layer of our model. (a) Raw data; (b) outputs from CONV-Net; (c) outputs from ACOVP layer; (d) outputs from SPDC-Net. Red and blue dots represent the target and nontarget trials, respectively.

We use t-distributed stochastic neighbor embedding (t-SNE) technique [33] to project the data with different representations into a 3-dimensional space for visualization. The t-SNE projections of the raw data and the outputs from each layer of our model are plotted in Figures 9 and 10 for 100% of full set data (subject 8) and 25% of full set data (subject 3), respectively. As can be seen from Figures 9(a) and 10(a), the target and nontarget trials overlap significantly in the raw representation. Although Figures 9(b) and 10(b) have revealed the aggregation of data from different classes into clusters with the convolutional feature representation, it is still hard to well separate target and nontarget trials from each other. Subsequently, more compact clusters are obtained with the midlevel augmented covariance representation than with the convolutional feature representation (see Figures 9(c) and 10(c)), indicating that the intraclass variations are reduced. After the geometric processing with SPDC-Net, Figures 9(d) and 10(d) have shown two coherent clusters with clearly visible interclass discriminability. In a word, all these three stages used in sequence contribute to the discrimination of target and nontarget FRP trials.

### 5.3. Comparison Results between Different Components for Our Model

#### 5.3.1. Effect of the Fully Temporal Convolutional Subnetwork

As shown in Section 5.2.1 and Section 5.2.2, our model with the fully temporal convolutional subnetwork has generally demonstrated better performance compared to SPDNet that takes the raw EEG trial for building the augmented covariance. To further study the effect of the fully temporal convolutional layers in our model, we have implemented a model that takes the feature maps from the second convolutional block of EEGNet for building the augmented covariance, instead of the first fully temporal convolutional block of EEGNet as in our model. The comparison results are summarized in Table 4. It leads to significantly inferior performance by adopting the feature maps from the second convolutional block of EEGNet for building the augmented covariance, compared to adopting those from the first block as in our approach (second block-first block: −0.0132 ± 0.0180, *p* = 0.0488). Such results have revealed that the temporal information is likely to be ruined for the feature maps from the second block of EEGNet, whereas it is preserved by those from the first block of EEGNet, validating the usage of the fully temporal convolutional subnetwork in our model.

#### 5.3.2. Effect of the Augmented Covariance Pooling Layer

We have examined the influence of adopting the covariance pooling layer instead of the augmented covariance layer in our model on its performance. To this end, we fix the CONV-Net and the SPDC-Net, but replacing the ACOVP layer with the covariance pooling (COVP) one. Results shown in Table 5 have revealed the advantage of ACOVP over COVP (COVP-ACOVP: −0.0466 ± 0.0524), with statistically significant difference (*p* < 0.05). This has confirmed the benefit of augmented covariance pooling in our method, which embeds the useful temporal ordering information when building the midlevel SPD representation (i.e., the augmented covariance), compared to the covariance pooling.

## 6. Discussion

In the deep learning framework, this study has proposed a novel classification model to discriminate the brain response to fixation onto target from that onto nontarget with single-trial FRPs. We evaluate the proposed approach against the baseline shallow models as well as state-of-the-art deep models consisting of first-order only or second-order only subnetworks. The experimental results obtained on different amounts of data show that the compared ones are consistently outperformed by our model, in which the first-order and second-order subnetworks are built in sequence. Toward a deep understanding of the proposed approach, the model architecture and pipeline components design choices are discussed below, followed by limitations and future work.

### 6.1. Model Architecture

#### 6.1.1. Deep Models vs. Baseline Shallow Models

The baseline first-order models (PCA + LDA and xDAWN + LDA) and second-order ones (MDRM and TSLDA) are widely adopted in previous studies [10, 11, 19] for classification of single-trial ERPs. However, our experimental results on full set of data have demonstrated that the deep models statistically outperform these shallow models by a significant margin (see Table 3). Such a performance discrepancy is in accordance with the reports in recent ERPs classification studies with deep models [16], verifying that the shallow models are less effective than deep ones in discriminating single-trial target and nontarget FRPs.

#### 6.1.2. Our Model vs. State-of-the-Art Deep Models

On one hand, the compared state-of-the-art first-order deep models, i.e., DeepConvNet and EEGNet, automatically learn discriminative first-order statistics from multidomains (time, frequency, and electrode location). Nevertheless, none of them are able to leverage the second-order information in the data. On the other hand, the compared state-of-the-art second-order deep model SPDNet could effectively extract the second-order statistics through deep Riemannian geometric processing. However, it is found in our study that utilizing the raw EEG trial to build the SPD matrix representation, on which the deep Riemannian geometric processing is conducted, suffers from low SNR due to the absence of first-order frequency-specific spatial filtering operations, in particular for cases lacking sufficient data. In contrast, our model takes advantage of the first-order frequency-specific spatial filtering with convolution operations and the following second-order deep Riemannian geometric processing.

As shown in the experimental results (Section 5.2.2), the proposed model has significantly outperformed the compared state-of-the-art first-order and second-order deep models on different amounts of data. In particular, with only 25% of data, our model has obtained comparable performance to that of EEGNet and DeepConvNet trained on 100% of data, whereas the performance of data-intensive SPDNet has degraded dramatically. All the findings in this study indicate that the proposed model is effective in learning a discriminative representation for single-trial classification of FRPs, and it is robust in the presence of limited data, thus making the approach appealing to use in practice.

Note that the state-of-the-art deep models taking the time-frequency representation of EEG scalp channels as input (e.g., [34]) have not been compared, since the model input often involves a significantly high dimensionality and thus requires intensive data to train the model, making them difficult to learn an effective feature representation with limited data as in our study.

### 6.2. Pipeline Component Design Choices

According to results shown in Section 5.3.1, the abstraction in time scale by the second convolution block of EEGNet is harmful to produce a good SPD representation for characterizing the intrinsic temporal structure of FRPs. Therefore, we have adopted the first convolutional block of EEGNet rather than the two convolutional blocks of EEGNet for the component CONV-Net in our model. Moreover, as the results in Section 5.3.2 suggest, the temporal ordering information preserved in the ACVOP layer appears to be crucial for producing improved performance. To sum up, in order to capture discriminative and robust high-level feature representations for single-trial classification of FRPs, the temporal ordering information should be well preserved in both the low-level and midlevel representations within our proposed feature learning pipeline.

### 6.3. Limitations and Future Work

The main limitation of current approach is that the components in the pipeline of our model are trained independently. The outputs from the trained fully temporal convolutional subnetwork CONV-Net are used to build the augmented covariance SPD representation, on which the SPD matrix learning and classification subnetwork SPDC-Net is trained without fine-tuning CONV-Net through backpropagations. Future work will investigate whether end-to-end joint training of CONV-Net and SPDC-Net leads to further performance improvements.

The guided visual search task employed in this study is still a well-controlled paradigm, and we are currently developing a more natural one, i.e., a free viewing search task, where a subject is free to fixate onto any space of the screen without the guiding circle. Moreover, this paper has only studied the technical foundation for measuring the target-perceptual ability with the EEG-based brain imaging technique, i.e., how to obtain a discriminative and robust feature representation for FRPs. In future, we plan to further apply the proposed feature representation learning method in early AD detection applications, where the target-perceptual ability of a subject is assessed with FPRs.

## 7. Conclusions

As a preliminary study of the technical foundation for measuring the target-perceptual ability using the EEG-based brain imaging technique, this paper proposes a novel method for extracting a good feature representation for FRPs, which could differentiate between the fixation onto target and the fixation onto nontarget from single-trial FRPs. Firstly, by using 1D convolution along the time axis and 1D convolution along the channel axis, the SNR of local features is enhanced by the convolutional operations, which act as tempo-spatial filtering on raw EEG signals. Secondly, the augmented covariance pooling layer builds an SPD matrix representation, encoding the second-order statistics (e.g., correlations) for the convolutional feature maps. Lastly, the deep Riemannian network further extracts a maximally separable global representation for classification from the augmented covariance matrix. In particular, the temporal ordering of FRPs is well preserved for the components in the pipeline, which is considered to be a valuable source of discriminant information. The experimental results obtained on different amounts of data show that our model has outperformed state-of-the-art models consisting of first-order only or second-order only subnetworks. Thereby, the discriminative and robust FRP representation extracted by our method may be potentially used for measuring the target-perceptual ability in early detection of AD.

## Figures and Tables

**Table 1:** Summary of FRP datasets for the ten participants.

Subject	#Nontarget	#Target	#Trials
1	717	115	832
2	616	95	711
3	436	82	518
4	660	108	768
5	739	106	845
6	802	98	900
7	735	104	839
8	834	120	954
9	605	78	683
10	659	109	768
Total	6803	1015	7818

**Table 2:** Output sizes of each stage in the pipeline.

Layer	CONV-Net		ACOVP	SPDC-Net			
	Conv-t + BN	Conv-S + BN + ELU		BiRe	BiRe	LogEig	Flatten
Output size	8×19×128	16×1×128	32×32	32×32	32×32	32×32	1024×1

**Table 3:** Performance (5-fold CV AUC) comparison on full set of data.

Method	S1	S2	S3	S4	S5	S6	S7	S8	S9	S10	Mean ± Std
PCA + LDA	0.8467	0.7596	0.8460	0.7678	0.8085	0.8324	0.8871	0.8733	0.7932	0.8353	0.8250 ± 0.0423
xDAWN + LDA	0.8531	0.7504	0.8711	0.8198	0.8366	0.8017	0.8808	0.8957	0.7971	0.8380	0.8344 ± 0.0439
DeepConvNet	0.9135	0.8603	0.9193	0.8456	0.9075	0.8634	0.9188	0.9071	0.8959	0.9167	0.8948 ± 0.0277
EEGNet	0.9307	0.8568	0.9214	0.8589	0.8970	0.8597	0.8999	0.9272	0.9188	0.9262	0.8997 ± 0.0305

MDRM	0.7901	0.6995	0.9170	0.7562	0.8858	0.8237	0.9377	0.8485	0.8996	0.9382	0.8496 ± 0.0813
TSLDA	0.9298	0.8587	0.9059	0.6931	0.8619	0.8118	0.9464	0.8946	0.7898	0.9270	0.8619 ± 0.0783
SPDNet	0.9453	0.8755	0.9307	0.8524	0.9366	0.8713	**0.9480**	0.9091	0.8999	0.9251	0.9094 ± 0.0336

Ours	**0.9521**	**0.8907**	**0.9519**	**0.8895**	**0.9400**	**0.9236**	0.9428	**0.9324**	**0.9451**	**0.9489**	0.9317 ± 0.0236

**Table 4:** Performance (5-fold CV AUC) difference between the midlevel SPD matrices, respectively, aggregated by outputs from the first and the second convolutional blocks of EEGNet.

	S1	S2	S3	S4	S5	S6	S7	S8	S9	S10	Mean±Std
Second block-first block (ours)	−**0.0306**	**−0.0032**	0.0095	**−0.0195**	**−0.0209**	**−0.0490**	0.0070	**−0.0088**	**−0.0163**	**−0.0002**	**−0.0132**±**0.0180**

**Table 5:** Classification performance (5-fold CV AUC) comparison between the augmented covariance pooling and the covariance pooling.

	S1	S2	S3	S4	S5	S6	S7	S8	S9	S10	Mean±Std
COVP-ACOVP (ours)	**−0.0814**	**−0.0209**	**−0.0094**	**−0.0423**	**−0.0610**	**−0.1749**	0.0106	**−0.0126**	**−0.0280**	**−0.0462**	−0.0466 ± 0.0524

## Data Availability

The FRP datasets are available from the corresponding author upon a reasonable request.
